# Crystallographic and molecular dynamics simulation analysis of *Escherichia coli* dihydroneopterin aldolase

**DOI:** 10.1186/2045-3701-4-52

**Published:** 2014-09-02

**Authors:** Jaroslaw Blaszczyk, Zhenwei Lu, Yue Li, Honggao Yan, Xinhua Ji

**Affiliations:** Macromolecular Crystallography Laboratory, National Cancer Institute, Frederick, MD 21702 USA; Department of Biochemistry and Molecular Biology, Michigan State University, East Lansing, MI 48824 USA; Centre of Molecular and Macromolecular Studies, Polish Academy of Sciences, Sienkiewicza 112, 90-363 Lodz, Poland; Department of Biochemistry and Center for Structural Biology, Vanderbilt University School of Medicine, Nashville, Tennessee 37232 USA

**Keywords:** Dihydroneopterin aldolase, DHNA, Structure, Dynamics, Catalysis

## Abstract

**Background:**

Dihydroneopterin aldolase (DHNA) catalyzes the conversion of 7,8-dihydroneopterin to 6-hydroxymethyl-7,8-dihydropterin and also the epimerization of DHNP to 7,8-dihydromonapterin. Previously, we determined the crystal structure of *Staphylococcus aureus* DHNA (SaDHNA) in complex with the substrate analogue neopterin (NP). We also showed that *Escherichia coli* DHNA (EcDHNA) and SaDHNA have significantly different binding and catalytic properties by biochemical analysis. On the basis of these structural and functional data, we proposed a catalytic mechanism involving two proton wires.

**Results:**

To understand the structural basis for the biochemical differences and further investigate the catalytic mechanism of DHNA, we have determined the structure of EcDHNA complexed with NP at 1.07-Å resolution [PDB:2O90], built an atomic model of EcDHNA complexed with the substrate DHNP, and performed molecular dynamics (MD) simulation analysis of the substrate complex. EcDHNA has the same fold as SaDHNA and also forms an octamer that consists of two tetramers, but the packing of one tetramer with the other is significantly different between the two enzymes. Furthermore, the structures reveal significant differences in the vicinity of the active site, particularly in the loop that connects strands β3 and β4, mainly due to the substitution of nearby residues. The building of an atomic model of the complex of EcDHNA and the substrate DHNP and the MD simulation of the complex show that some of the hydrogen bonds between the substrate and the enzyme are persistent, whereas others are transient. The substrate binding model and MD simulation provide the molecular basis for the biochemical behaviors of the enzyme, including noncooperative substrate binding, indiscrimination of a pair of epimers as the substrates, proton wire switching during catalysis, and formation of epimerization product.

**Conclusions:**

The EcDHNA and SaDHNA structures, each in complex with NP, reveal the basis for the biochemical differences between EcDHNA and SaDHNA. The atomic substrate binding model and MD simulation offer insights into substrate binding and catalysis by DHNA. The EcDHNA structure also affords an opportunity to develop antimicrobials specific for Gram-negative bacteria, as DHNAs from Gram-negative bacteria are highly homologous and *E. coli* is a representative of this class of bacteria.

## Background

**Figure 1:**
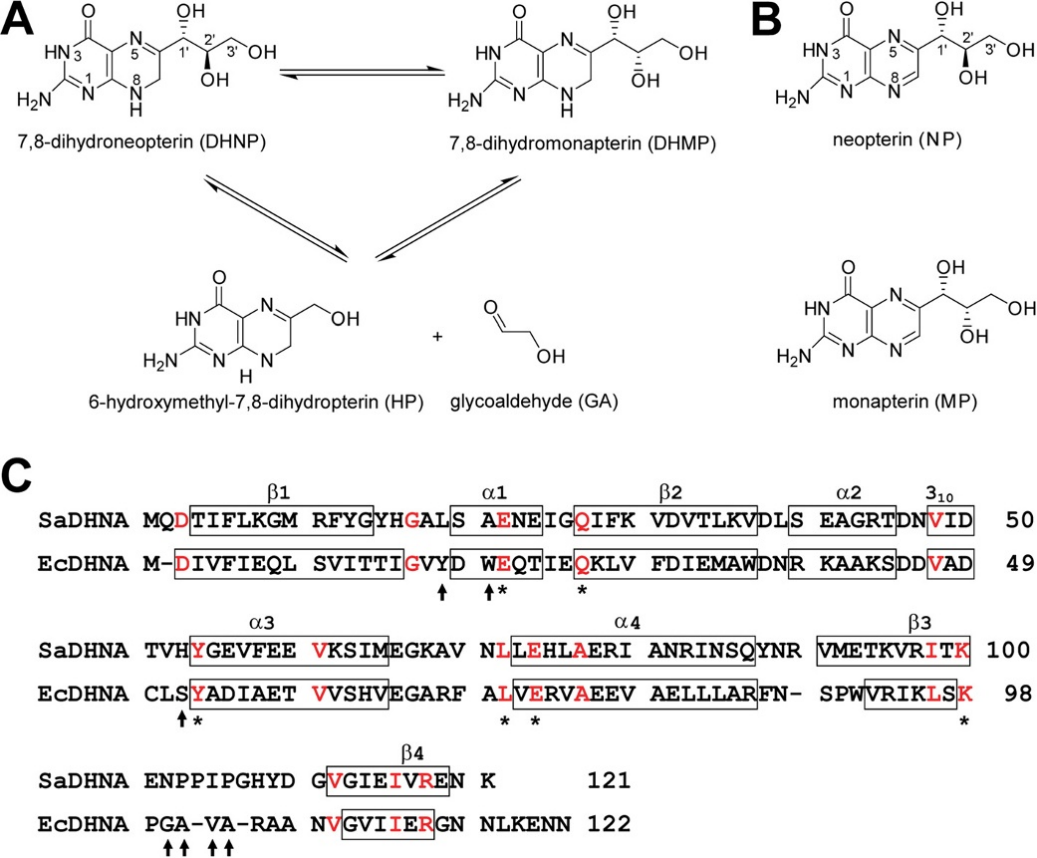
**Reaction and sequence of DHNA. (A)** Reactions catalyzed by DHNA. **(B)** Chemical structures of NP and MP. **(C)** Structure-based sequence alignment of Gram-positive SaDHNA [Swiss-Prot:P56740; PDB:2NM3] and Gram-negative EcDHNA [Swiss-Prot:P0AC16; PDB:2O90]. Secondary structural elements are outlined with boxes. The conserved amino acid residues among all DHNA sequences are highlighted in red, while the sequence variations that have significant impact on ligand binding are indicated with arrows in black.

Dihydroneopterin aldolase (DHNA) catalyzes the conversion of 7,8-dihydroneopterin (DHNP) to 6-hydroxymethyl-7,8-dihydropterin (HP) with the generation of glycoaldehyde [1] (Figure 1). This is a committing step in the folate pathway because HP is the first compound in the pathway used only for the biosynthesis of folate cofactors. DHNA functions as a homooctamer. The enzyme is a unique aldolase in that it requires neither metal ions nor the formation of a Schiff base between the enzyme and the substrate for catalysis [1]. Furthermore, the enzyme also catalyzes the epimerization of DHNP to 7,8-dihydromonapterin (DHMP) at a significant rate, but the biological function of DHMP is not clear [2].

Folate cofactors are essential for life [3]. Mammals obtain folates from their diet because they cannot synthesize folates *de novo* but have an active transport system. In contrast, most microorganisms must synthesize folates *de novo* because they cannot take folates from their environments due to the lack of an active transport system [4]. Therefore, the folate biosynthetic pathway has been one of the principal targets for developing antimicrobial agents [5–9]. Among the folate pathway enzymes, the four enzymes in the mid pathway are particularly attractive because they are absent in mammals: DHNA, 6-hydroxymethyl-7,8-dihydropterin pyrophosphokinase (HPPK), dihydropteroate synthase (DHPS), and dihydrofolate synthase (DHFS). DHPS is the target of sulfa drugs, the clinical use of which marks the beginning of the modern era of antimicrobial chemotherapy [10]. The multiple targets afforded by this pathway also provide opportunities to develop antibiotics with synergetic effects. For example, in clinical use, sulfonamides, which target DHPS, are combined with trimethoprim, an antibiotic targeting DHFS, the last enzyme in the folate pathway [10].

Interestingly, DHNAs from Gram-positive and Gram-negative bacteria have some unique sequence motifs [11]. The sequence identities between enzymes from Gram-positive bacteria range from 39% to 45% and those between Gram-negative bacteria are 49-91%, but the identities between Gram-positive and Gram-negative bacterial enzymes are <30% [11]. Many differences between the amino acid sequences of DHNAs from Gram-positive and Gram-negative bacteria are at or near the active center. In accordance with the significant differences between their sequences, biochemical studies have shown that EcDHNA and SaDHNA have significantly different ligand binding and catalytic properties [11–13].

To date, crystal structures have been reported for DHNAs from Gram-positive bacteria *S. aureus* [14–16], *Mycobacterium tuberculosis* (MtDHNA) [17], *Streptococcus pneumoniae* [18], and the plant *Arabidopsis thaliana* [19]. The active site of the enzyme has been identified by the protein-product (HP) structures of SaDHNA [PDB:2DHN] [14] and MtDHNA [PDB:1NBU] [17]. The structural information about the critical interactions between DHNA and the trihydroxypropyl moiety of the substrate, which undergoes bond cleavage and formation, has been revealed by our structures of SaDHNA in complex with neopterin (NP) [PDB:2NM2] and with monapterin (MP) [PDB:2NM3], respectively [16]. NP and MP are excellent inhibitors for SaDHNA, because the only difference between these inhibitors and the corresponding substrates is that the single bond between C7 and N8 in the substrates is replaced by a double bond in the inhibitors (Figure 1). The crystal structures of SaDHNA in complex with NP or MP have provided important insights into the catalytic mechanism of the enzyme [16].

No crystal structure has been reported for DHNAs from Gram-negative bacteria. Because *E. coli* is a representative of Gram-negative bacteria and EcDHNA has been well characterized biochemically [11], we have determined the crystal structures of EcDHNA in complex with the substrate analogue NP (EcDHNA:NP) [PDB:2O90]. Based on this crystal structure, we have built an atomic model of the enzyme in complex with the substrate DHNP (EcDHNA:DHNP) and performed molecular dynamics (MD) simulation of the enzyme:substrate complex. The results provide insights into the mechanism of DHNA catalysis, the structural basis of biochemical differences between SaDHNA and EcDHNA, and valuable information for structure-based design of novel antimicrobial agents.

## Results

### Overall structure of the EcDHNA:NP complex

The EcDHNA:NP structure has been determined at 1.07-Å resolution. The asymmetric unit of the structure contains one DHNA polypeptide, one NP molecule, and 279 water molecules. Thus, the octamer of EcDHNA:NP contains eight identical active sites. Seven residues at the C-terminus (Asn116-Asn122) are not observed and thus presumably disordered. Met1 exhibits three conformations of equal probabilities; 20 residues (Ile3, Gln8, Ser10, Val17, Tyr18, Asp19, Lys27, Asp31, Glu33, Arg39, Ser62, Arg68, Leu82, Arg93, Ile94, Ser97, Pro99, Gly100, Ala101, and Glu113) assume two conformations.

### Interactions between EcDHNA and NP

**Figure 2:**
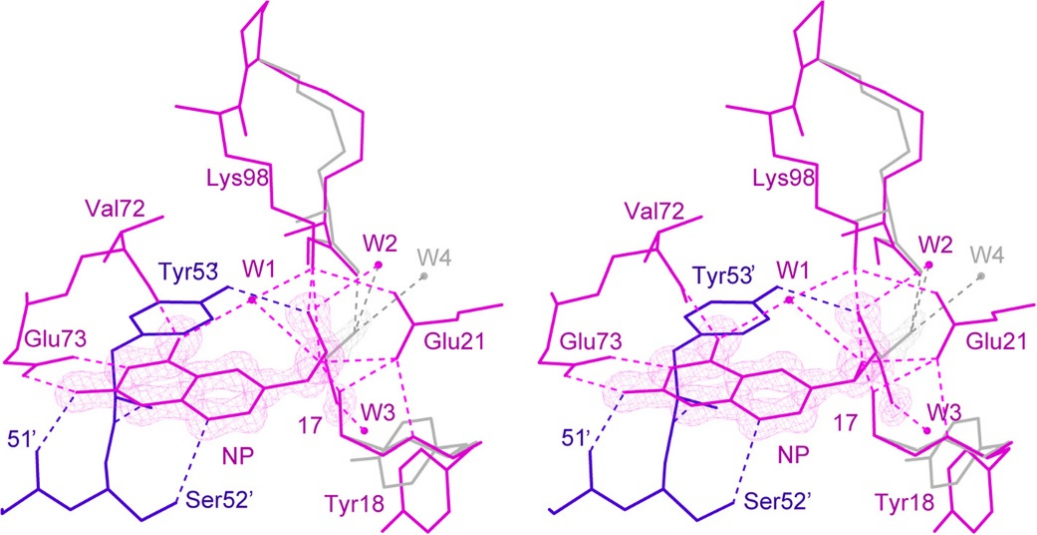
**Stereoview showing the active center structure of EcDHNA:NP.** The 2*F*_o_ - *F*_c_ electron density (net in purple) contoured at 2.5 *σ* for the NP molecule and at 1.2 *σ* for the disordered portion of the trihydroxypropyl moiety (net in gray). Residues colored in blue are from the symmetry-related molecule that forms the active center with the primary molecule. The illustration was made using MolScript [20].

The bound NP and its most important interactions with EcDHNA are illustrated in Figure 2. The trihydroxypropyl tail of NP assumes two conformations, with the 3′-hydroxyl group in significantly different positions. NP is surrounded by residues Ile15-Tyr18, Trp20, Glu21, Gln26, Ala70-Glu73, Lys98, Gly100-Val102, and Val108-Val110 from one subunit and Val4′, Val47′, and Cys50′-Asp55′ from the adjacent subunit (denoted by the prime symbols). The pteridine ring of NP is stacked on the phenol ring of Tyr53′, and other important interactions include hydrogen bonds to the side-chain groups of Glu73 and Ser52′, the main-chain groups of Leu72, Leu51′, and Tyr53′, and a water molecule (Figure 2). The water molecule is also hydrogen bonded to the main-chain carbonyl oxygen of Ala70 and the amino group of Lys98. The 1′-hydroxyl group of the trihydroxypropyl tail forms one hydrogen bond each to the main-chain NH of Val17 and the amino group of Lys98 and a bifurcated hydrogen bond to the carboxyl group of Glu21. The 2′-hydroxyl group is hydrogen bonded to both the amino group of Lys98 and the phenol group of Tyr53′. In one conformation, the 3′-hydroxyl group is hydrogen bonded to the main-chain carbonyl oxygen of Ala101, and in the other conformation, it has no direct interaction with the protein.

### Comparison with the SaDHNA structure

**Figure 3:**
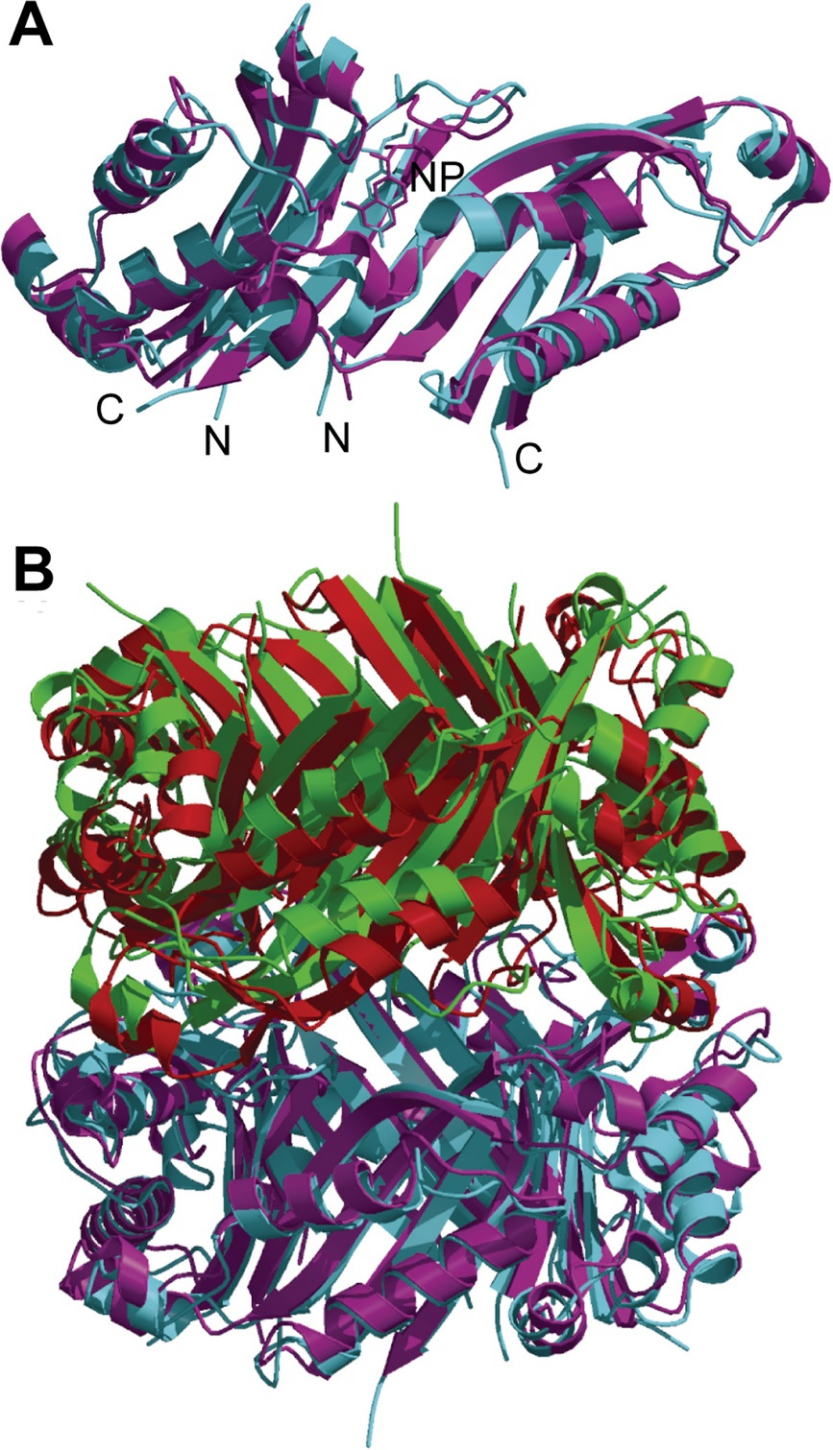
**Folding and packing comparison between EcDHNA:NP and SaDHNA:NP. (A)** Folding comparison between the two structures. **(B)** Packing comparison between the two structuers. In panel **A**, EcDHNA and SaDHNA are in magenta and cyan, respectively. In panel **B**, the two tetramers of EcDHNA are in magenta and red, and those of SaDHNA are in cyan and green. The illustration was made using MolScript [20] and Raster3D [21, 22].

The conformation of EcDHNA shows significant differences in comparison with those of other species. Because EcDHNA and SaDHNA are the best characterized among DHNAs [2, 11, 13, 14, 16] and, furthermore, *E. coli* and *S. aureus* are representatives of Gram-negative and Gram-positive bacteria, respectively, we compared the structures of these two enzymes. The root-mean-square deviation for main-chain atoms (RMSD) between the EcDHNA in the asymmetric unit of EcDHNA:NP and the four SaDHNA polypeptide chains in the asymmetric unit of SaDHNA:NP ranges 1.03-1.09 Å. The most significant differences are found between the active sites of the two enzymes (Figure 3A), particularly in the loop that connects strands β3 and β4. The conformational difference in the loop is obviously due to the significant sequence differences in this region (Figure 1). In SaDHNA, it consists of 11 residues, including three prolines, whereas in EcDHNA, it contains nine residues, including only one proline. The significance of these conformational differences will be discussed below.

The functional assembly of DHNA is an octamer, which consists of two stacked doughnut-shaped tetramers. The RMSD between the functional octamers of EcDHNA and SaDHNA reaches 1.67 Å, which is substantially larger than those between two single subunits. The large RMSD is due to the difference in the packing between the two tetramers (Figure 3B), which is mainly due to an 8° rigid body rotation. The indole rings of the Trp20 residue in one tetramer stack on those of the corresponding Trp residues in the other tetramer (not shown), and therefore, the Trp substitution, corresponding to Ala21 in SaDHNA, may play a role in stabilizing the EcDHNA octamer.

**Figure 4:**
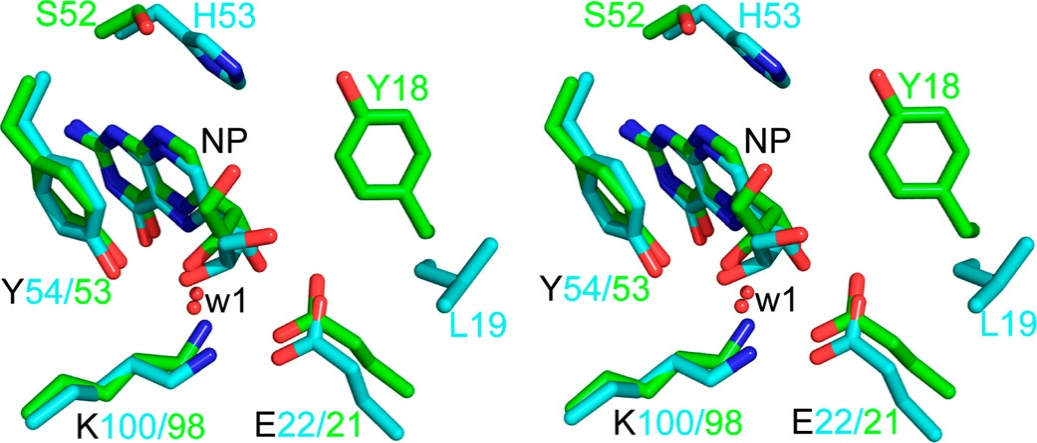
**Stereoview showing the active site comparison between EcDHNA:NP and SaDHNA:NP.** The NP molecules and amino acid residues are shown as stick models in atomic color scheme (carbon in green for EcDHNA and in cyan for SaDHNA, nitrogen in blue, and oxygen in red). The illustration was made using PyMOL (Delano Scientific, San Carlos, CA, USA).

**Figure 5:**
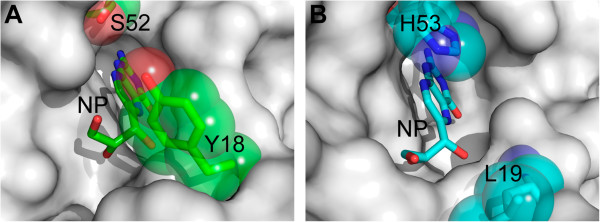
**Molecular surface representations for the active site in the EcDHNA:NP and SaDHNA:NP complexes. (A)** Active site structure in the EcDHNA:NP complex. **(B)** Active site structure in the SaDHNA:NP complex. The NP molecules and two variable amino acid side-chains are shown as stick models in atomic color scheme (carbon in green for EcDHNA and in cyan for SaDHNA, nitrogen in blue, and oxygen in red). The side-chains are also highlighted with transparent atomic surfaces. The illustration was made using PyMOL (Delano Scientific, San Carlos, CA, USA).

Biochemical studies have shown that the kinetic parameters for EcDHNA and SaDHNA are significantly different [11]. Among many sequence variations, seven amino acid substitutions (Figure 1C) occur in the vicinity of the active site, among which two are shown in Figure 4, Tyr18 and Ser52 in EcDHNA, corresponding to Leu19 and His53 in SaDHNA, respectively. These two substitutions may have greatest impact on the binding properties of the enzymes. The combination of Leu19 and His53 in SaDHNA result in a wide open exit to the active site, whereas the Tyr18 and Ser52 in EcDHNA, especially Tyr18, largely block the active-site exit (Figure 5). Furthermore, the hydroxyl group of Ser52 can form a hydrogen bond with reduced or oxidized pterin compounds, because the hydroxyl group can function as either a hydrogen bond donor or an acceptor. The hydrogen bond between the hydroxyl of Ser52 and an oxidized pterin compound may be even stronger than that with the corresponding reduced pterin compound, because the *K*_d_ value of EcDHNA for the oxidized HP is significantly lower than that for HP [11]. The *K*_d_ values of SaDHNA for the paired pterin compounds are the same [11], because the group at position 8 of the pteridine moiety has no hydrogen bond interaction with the protein [14]. These structural features are consistent with the significantly lower *K*_d_ values for the binding of NP, MP, HP, and oxidized HP by EcDHNA when compared with the *K*_d_ values for the binding of these ligands by SaDHNA [11]. In particular, the *K*_d_ value of SaDHNA for oxidized HP (24 μM) is 240 times that of EcDHNA (0.10 μM). The lower *K*_d_ values of EcDHNA for the pterin compounds are due to smaller dissociation rate constants [11]. The dissociation rate constant for the product HP is only 0.26 s^-1^, which is the rate-limiting step in the EcDHNA-catalyzed conversion of DHNP to HP, whereas the corresponding dissociation rate constant in the SaDHNA-catalyzed reaction is 14 s^-1^. The slower dissociation of the product HP in EcDHNA is probably due to the need of breaking the additional hydrogen bond with the hydroxyl group of Ser52 and the obligatory movement of the Tyr18 phenol ring.

### Dynamic properties of the enzyme-substrate complex EcDHNA:DHNP

**Figure 6:**
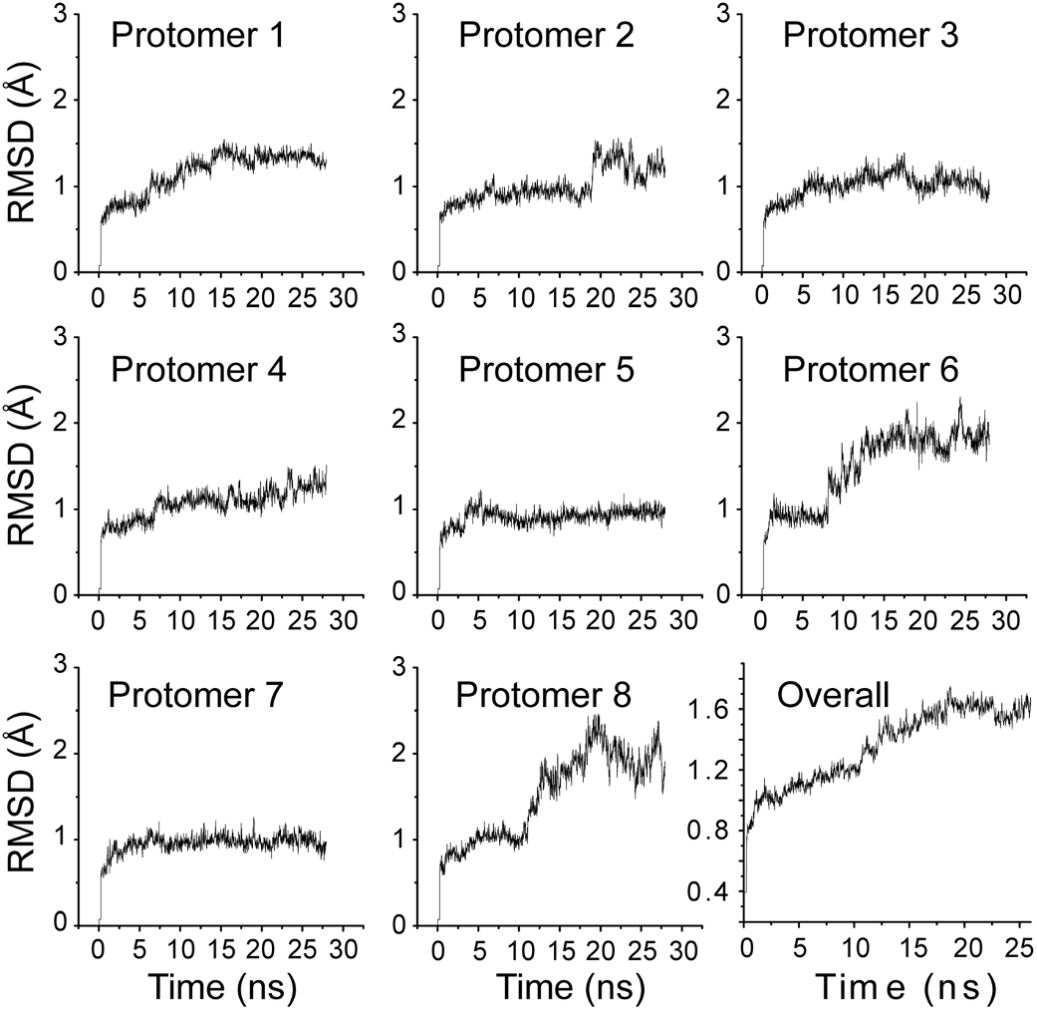
**RMSD (for main-chain atoms) of individual EcDHNA protomers in MD simulation of the enzyme:substrate complex.** The first eight panels are the RMSD of eight EcDHNA protomers based on the superposition of individual protomers. Panel 9 is the RMSD of the EcDHNA octamer based on the superposition of the octamer.

Because many residues at or near the active center assume multiple conformations in the high-resolution EcDHNA crystal structure, we were interested in and investigated the dynamic properties of the enzyme-substrate complex EcDHNA:DHNP by MD simulation. The MD trajectory of the 27-ns MD simulation is stable, with the RMSD from the crystal structure rising quickly to ~1 Å and stabilized at ~1.6 Å (Figure 6, panel 9). The magnitude of the RMSD of the 27-ns MD simulation of the EcDHNA-substrate complex is similar to those of the 2-ns MD simulations of the apo SaDHNA and the SaDHNA-product complex [23].

To assess whether the motions of individual protomers are independent, we calculated the RMSD of each protomer (Figure 6). The RMSD plots of protomers 3, 5, and 7 are similar, rising quickly to and staying at ~1 Å. The RMSD plots of protomers 6 and 8 are similar, with the RMSD values increasing in two stages, first rising to and staying at ~1 Å and then rising to and staying at ~2 Å. It is worthy to note that the transition between these two stages is different for the two protomers, with one much earlier than the other. The RMSD plots of the other three protomers are in between. The differences in the RMSD plots and their uncorrelated nature indicate that the motions of individual protomers are largely independent, in consistence with the uncooperative binding of NP and DHNP to the enzyme observed in biochemical experiments [11]. Consequently, the analyses of the MD data are based on the behaviors of individual protomers rather than the octomer as a whole. The MD data are effectively composed of eight 27-ns trajectories, equivalent to an aggregate simulation time of 216 ns, and the results below are the average of the eight trajectories.

**Figure 7:**
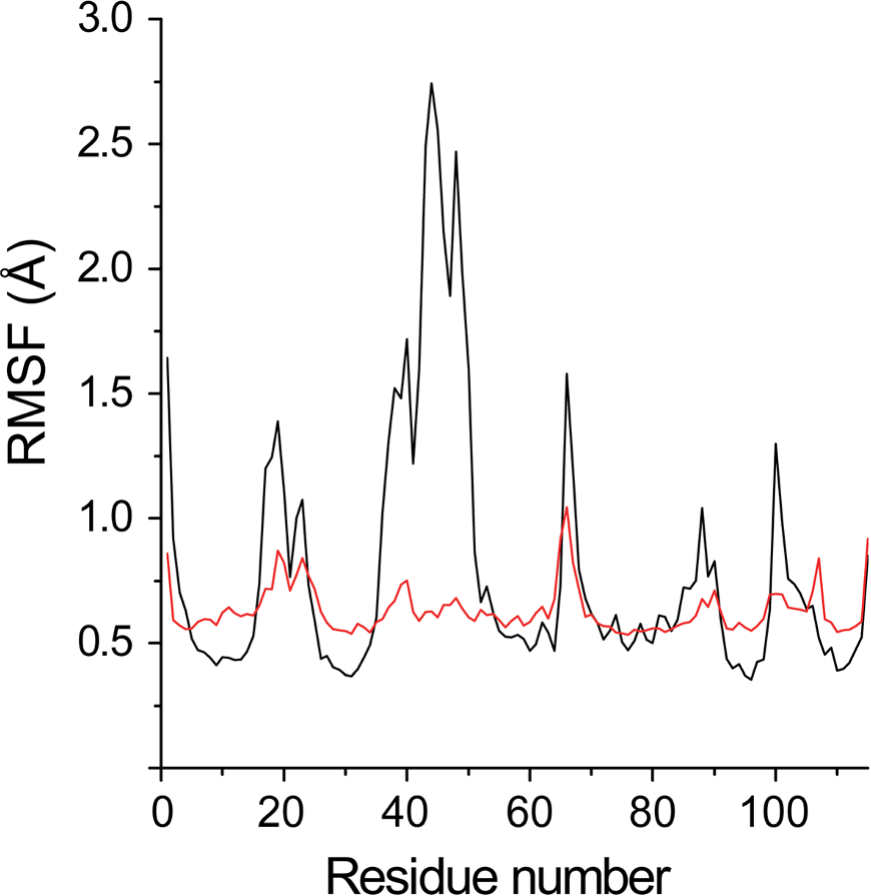
**RMSF of the Cα atoms of EcDHNA.** Black, calculated from the trajectory of the MD simulation of the substrate complex; red, calculated from the B factors of the crystal structure of the inhibitor complex for comparison.

The flexibility of EcDHNA is assessed by the analysis of the Cα root-mean-square fluctuation (RMSF) of each residue (Figure 7) calculated based on the superposition of individual protomers as we have done in the analysis of the MD trajectories of SaDHNA [23]. The overall RMSF from the MD simulation at 300 K is significantly larger than that from the B factors of the crystal structure derived from the diffraction data obtained at 100 K, suggesting that the protein is much more flexible than indicated by the crystallographic B factors. Based on the RMSF analysis, two long regions of residues show significant flexibility, residues 16-24 and 35-53, the functional significance of which will be discussed in a later section. Besides the terminal residues, additional residues with significant motion include residues 65-70, 85-91, and 99-106.

**Figure 8:**
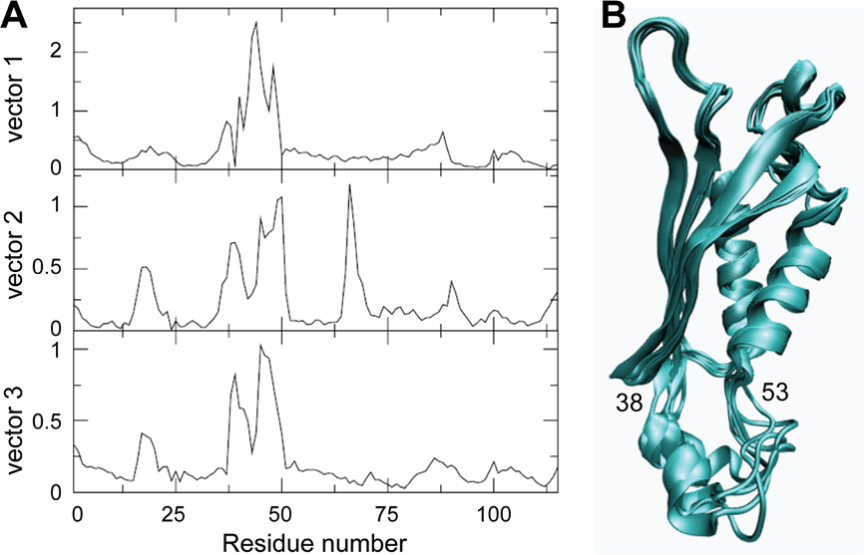
**Principal component and clustering analyses. (A)** Projection of Cα-atom RMSF in the first three principal components. **(B)** Overlay of representative structures from cluster analysis. The segment of the protein with the largest deviation in panel **B** is labelled with the beginning and ending residue numbers.

The flexibility of EcDHNA is further characterized by Principal component analysis (PCA) to identify the dominant modes of fluctuation. The results show that the first three modes accounts for 52.5% of total fluctuation, the first five modes account for 63.8%, and the first ten modes account for 74.8%, indicating that the major motions of EcDHNA can be described by the first several modes of fluctuation. Figure 8A shows the Cα atom RMSF projections onto the first three principal component eigenvectors. It is clear that motion in the first principal component mode (vector 1) is dominated by residues 38-53; motion in the second principal component mode (vector 2) dominated by residues 15-25, 38-53, and 65-70; and motion in the third principal component mode (vector 3) dominated again by residues 38-53. Since the motion of residues 38-53 are prominent in all three principal component modes and these principal component vectors are orthogonal, these residues must have complex motional behavior. To illustrate this complex motional behavior, we grouped the snapshots of the whole trajectory into five clusters as illustrated in Figure 8B. The representatives of the five clusters are very similar to each other except the region encompassing residue 38-53. Even the small α-helix (residue 37-45) within this region shows different conformations among those representative structures: It can shorten or bend.

### Interactions between the substrate DHNP and the enzyme

**Figure 9:**
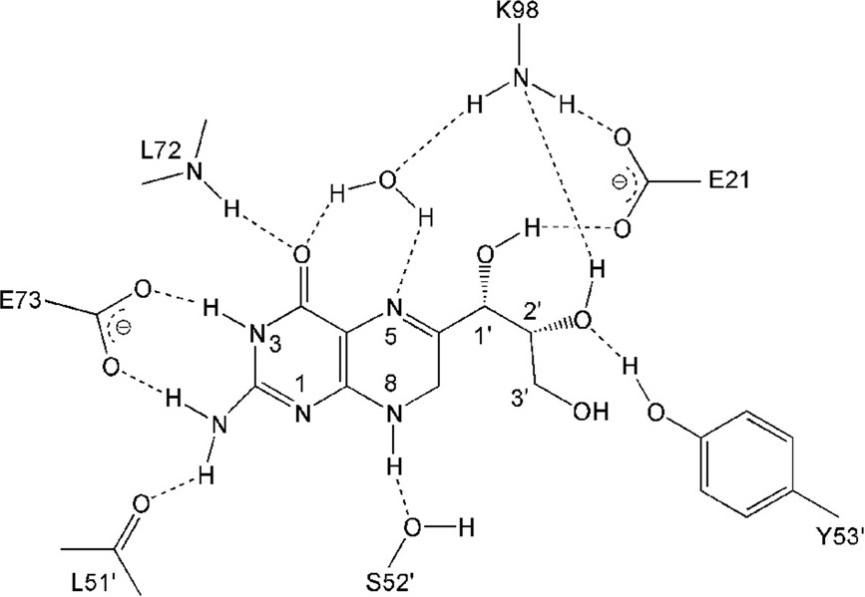
**Hydrogen bonding between substratrate and enzyme observed in MD simulation of the enzyme:substrate compplex.** Hydrogen bonds are shown in dashed lines. Only hydrogen bonds with larger than 50% occurrence were drawn. A schematic diagram showing how DHNP is cleaved into HP and GA is presented in Figure 1A.

Since hydrogen bond interactions are important for not only substrate binding but also catalysis by DHNA, we focused our analysis of the interaction between the substrate and the enzyme on hydrogen bonds. The results are summarized in Table 1 and illustrated in Figure 9. The hydrogen bonds between the substrate DHNP and the enzyme EcDHNA suggested by the MD simulation are largely similar to those between the inhibitor NP and the enzyme EcDHNA in the crystal structure, indicating that the inhibitor NP is a good mimic of the substrate DHNP and the MD simulation indeed reflects the dynamics of the enzyme. However, the MD simulation data also provide some significant new information about the interactions between the substrate and the enzyme. First, the hydroxyl group of Ser52′ functions as a donor and forms a hydrogen bond with 8-NH of DHNP. Second, the hydrogen bond corresponding to that between 3′-hydroxyl of the inhibitor and the enzyme in the crystal structure is essentially nonexistent in the MD trajectory of the enzyme-substrate complex, observed in only 7.33% of the snapshots of the MD trajectory. Finally, some hydrogen bonds are much more persistent or stable than others. The most persistent hydrogen bonds between the substrate and the enzyme are those of the substrate with the carboxylate of Glu73 and the backbone amides of Leu51′ and Leu72, observed in >95% of the snapshots of the MD trajectory. The hydrogen bond between N1 of DHNP and the backbone NH of Tyr53′ is weak, with an occurrence of only ~16%. Based on the hydrogen bond occurrence data (Table 1), the pterin portion of the substrate is fixed in the active site, the 1′- and 2′-hydroxyl groups have some motions, and the 3′-hydroxyl group is flexible. The flexibility of 3′-hydroxyl allows both DHNP and DHMP to serve as substrates for the enzyme.

**Table 1:** **Hydrogen bonds between DHNP, the catalytic water, and DHNA revealed by molecular dynamics simulation**

	occurrence	distance	EcDHNP:NP ^a^
	(%)	(Å)	(Å)
Leu72 - NH…O = C4	99.73	2.91 ± 0.14	2.94
WAT - OH… O = C4	82.2	2.94 ± 0.20	2.79
Glu73 - OE1…H - N3	100	2.79 ± 0.09	2.76
Glu73 - OE2…H - NC2	99.74	2.84 ± 0.13	2.73
Leu51′ - O…H - NC2	95.94	2.93 ± 0.15	2.85
Tyr53′ - NH…N1	16.04	3.33 ± 0.12	2.98
WAT - OH…N5	70.61	3.18 ± 0.19	3.31
Ser52′-OG…H - N8	88.82	3.03 ± 0.17	NA^b^
Glu21 - OE1…H - OC1′	80.59	2.69 ± 0.24	2.64
Val17 - NH…OC1′	80.15	2.98 ± 0.20	2.88
Lys98 - NZ… H - OC2′	76.54	2.95 ± 0.16	2.75
Tyr53′ - OH…OC2′	63.96	3.10 ± 0.19	2.61
Ala101 - O…H - OC3′	7.33	3.00 ± 0.24	2.90
Lys98 - NZH…WAT	91.5	2.98 ± 0.17	2.87

^a^The corresponding hydrogen bonds in the crystal structure of EcDHNP:NP are listed for comparison. ^b^NA, not applicable.

## Discussion

### Insights into substrate binding and catalysis

DHNA is a special aldolase because it does not require a zinc ion or the formation of a Schiff base between the enzyme and the substrate for catalysis and generates an epimerization product at a significant rate. The hallmark of its catalytic power is general acid/base catalysis. Two proton wires have been proposed for DHNA catalysis [16]. The first proton wire consists of a conserved Lys residue, a catalytic water molecule, and 2**′**-OH and N5 of the substrate DHNP. This proton wire is revealed by the crystal structure of SaDHNA in complex with NP [PDB:2NM2] and is required for the cleavage of the C1**′**-C2**′** bond, which generates the enol intermediate. The importance of the conserved Lys residue, Lys98 in EcDHNA, for catalysis has been demonstrated by site-directed mutagenesis. The second proton wire consists of a conserved Tyr residue, Tyr53 in EcDHNA, the conserved Lys residue, the catalytic water, and C1**′** and N5 of the reaction intermediate. This proton wire is proposed based on a mutagenesis study of the conserved Tyr residue of DHNA [16]. The mutagenesis study has shown that the Tyr residue is not required for the generation of the reaction intermediate but for protonation of it to generate the product HP. The first proton wire is present in the crystal structure of EcDHNA in complex with NP and the atomic model of the complex of EcDHNA and the substrate DHNP reported here. The formation of the second proton wire needs significant local conformational adjustment. In consistence with this requirement, the segment of the protein containing Tyr53 (residues 38-53) is most flexible in the MD simulation with the highest RMSF. This flexibility may also allow the rotation of the product glycoaldehyde and generation of the epimerization product MP.

The MD simulation of the EcDHNA complex also shows that the substrate DHNP is anchored in the active site by four hydrogen bonds between the substrate (the groups at positions 2, 3, and 4) and the protein (backbone amides of Leu51′ and Leu72 and carboxylate of Glu73). This is manifested by the persistence of these hydrogen bonds, found with >95% of the snapshots of the MD simulation. Particularly, the carboxylate of Glu73 forms two hydrogen bonds, one with 1-NH and the other with 2-NH_2_, the former with a 100% of occurrence and the latter with a 99.7% of occurrence. Indeed, mutagenesis studies have shown that Glu73 is the most important residue for binding of the substrate DHNP, the inhibitor NP, and the product HP. In contrast, the hydrogen bonds between the trihydroxyl moiety of DHNP and the protein are transient, with occurrence in the range of 7.3 - 80.6%. In particular, the hydrogen bond between 3′-OH of the substrate and the backbone amide of Ala101 observed in the crystal structure is largely absent in the MD simulation, with an occurrence of only 7.3%. The transient nature of these hydrogen bonds, particularly the rare occurrence of the hydrogen bonding of 3′-OH, may allow the epimer DHMP serve as a good substrate as demonstrated by biochemical analysis [11].

The asynchronous motions of individual protomers observed in the MD simulation are also biochemically important. Although DHNA is a homooctomeric protein, it does not show cooperativity in binding substrate or other ligands. This lack of cooperativity is consistent with the asynchronous motions of individual protomers in the MD simulation.

### Implications for structure-based drug design

Infectious diseases are the leading causes of death and the main causes of premature death (0-44 years) [24]. Widespread and persistent antibiotic resistance has caused a worldwide health care crisis [5, 25, 26]. The crisis has been aggravated by the decisions by many major pharmaceutical companies to abandon or curtail their antibacterial programs for business reasons [27–29] and the fact that most new antibiotics are chemical modifications of existing antimicrobial agents [30]. These compounds act against old targets and are therefore less effective in dealing with widespread antibiotic resistance. New targets for the development of novel antimicrobial agents are thus urgently needed for combating the antibiotic crisis.

DHNA is an attractive target for developing new antibiotics, because the enzyme is in a biosynthetic pathway proven effective in developing antibiotics and is absent in human. Sanders and coworkers have reported the development of inhibitors against SaDHNA [15]. The fact that EcDHNA and SaDHNA have significant differences in their biochemical properties and active center structures, structure-based drug design must take into account the structure of EcDHNA for developing broad-spectrum antibiotics. On the other hand, DHNAs from Gram-negative bacteria are highly homologous, and so their active centers are expected to have very similar shapes and physicochemical characteristics. The structure of EcDHNA reported here offers an opportunity to develop antibiotics specific for Gram-negative bacteria.

## Conclusions

The EcDHNA:NP structure has been determined at 1.07 Å, the highest resolution among all of the DHNA structures reported to date. This crystal structure of the inhibitor complex and the MD simulation of the substrate complex EcDHNA:DHNP have provided important insights into substrate binding and catalysis. The substrate DHNP is anchored in the active site via four persistent hydrogen bonds. The transient nature of hydrogen bonding between the trihydroxyl moiety and the protein and the rare occurrence of hydrogen bonding with 3′-hydroxyl allow the pair of epimers DHNP and DHMP to serve as good substrates. The high flexibility of segments of the protein, particularly residues 38-53, may permit the switching from the first to the second proton wire during catalysis and the generation of the epimerization product. The asynchronous motions of individual protomers are consistent with the noncooperative binding of the substrate by DHNA. Between EcDHNA and SaDHNA, the two best characterized DHNAs, there are three outstanding structural differences. First, the packing of the two tetramers in the EcDHNA octamer is significantly different from that in the SaDHNA octamer due to a single mutation. Residue Trp20 in EcDHNA is an Ala in SaDHNA. The indole rings of the Trp20 residue in one tetramer stack on those of the corresponding Trp residues in the other tetramer, stabilizing the EcDHNA octamer. Second, the active site structures of the two enzymes, especially in the β3-β4 loop, are significantly different due to the difference in amino acid sequences. In SaDHNA, the loop is 11 residues in length, including three prolines, whereas in EcDHNA, it has nine residues, containing only one proline. Third, the entrance to the active site is significantly different in the two enzymes. The combination of Leu19 and His53 in SaDHNA result in a wide open entrance, whereas the Tyr18 and Ser52 in EcDHNA largely block the entrance. These structural differences provide the basis for the biochemical differences between the *S. aureus* and *E. coli* enzyme that represents Gram-positive and Gram-negative DHNA, respectively.

## Methods

### Crystallography

Cloning, expression, and purification of EcDHNA have been reported previously [11]. NP was purchased from the Schircks Laboratories. The crystals of EcDHNA:NP were obtained via co-crystallization using the hanging-drop technique at well-controlled room temperature (19 ± 1°C). The protein solution was mixed and incubated with the ligand prior to crystallization experiments. The drops contained equal volumes of protein and reservoir solutions. The protein solution contained 11 mg/mL protein and 25 mM NP in 10 mM Tris-HCl (pH 8.0). The well solution contained 4.0 M sodium formate (pH 7.0). Crystals reached the size of 0.25 × 0.35 × 0.60 mm in about three months.

The crystal was tetragonal (I422) and diffracted to 1.07-Å resolution. X-ray diffraction data were collected from a single crystal at 100 K with an ADSC Quantum-4 CCD detector mounted on the synchrotron beamline X9B at National Synchrotron Light Source, Brookhaven National Laboratory. Data processing was carried out with HKL2000 [31]. Data collection and processing details are summarized in Table 2.

**Table 2:** **X-ray data and refinement statistics for the EcDHNA:NP structure [PDB:2O90]**

Data	Overall	Last shell
Resolution range (Å)	30.0-1.07	1.11-1.07
Completeness (%)	95.1	64.6
Redundancy	9.1	
*I*/σ(*I*)	40.8	2.3
*R*_scaling_^*a*^	0.043	0.309
**Refinement**	**All data**	**I ≥ 2σ(I)**
Reflections used for refinement	53895	45617
Reflections used for *R*_free_	2832	2390
Number of least-squares parameters	9680	
Crystallographic *R* ^*b*^	0.130	0.118
*R*_free_^*c*^	0.150	0.137
**Structure**		
Number of protein atoms/average B factor (Å^2^)	1001/13.3	
Number of ligand atoms/average B factor (Å^2^)	23/13.2	
Number of water oxygen atoms/average B factor (Å^2^)	279/37.5	
RMSD from ideal geometry:		
Bond distances (Å)	0.019	
Angle distances (Å)	0.034	
Ramachandran plot:		
Most favored φ/ψ angles (%)	97.2	
Disallowed φ/ψ angles (%)	0.0	

^*a*^ *R*_scaling_ = Σ|*I*- < *I >* |/Σ*I*. ^*b*^Crystallographic *R* = Σ_hkl_ | |*F*_o_| - |*F*_c_| | / Σ_hkl_ |*F*_o_|. ^*c*^ *R*_free_ is calculated from 5% of data randomly chosen and not included in refinement.

The structure was solved by molecular replacement (MR) using the apo-DHNA structure [PDB:1DHN] as the search model after the solvent molecules were removed. The MR solutions were subjected to rigid body refinement, energy minimization, and grouped B-factor refinement followed by a difference Fourier synthesis, which revealed the locations of the ligand molecule.

The structure was refined using CNS [32] at the initial stage and SHELXL [33] till completion. Model building was carried out using the graphics package O [34]. The structure was refined with anisotropic temperature factors for non-hydrogen atoms, except for regions of increased mobility where the temperature factors were refined isotropically. For atoms with anisotropic temperature factors, the hydrogen atoms were built at idealized positions, with assigned isotropic temperature parameters equal to 1.2 times the equivalent isotropic temperature parameters of their parent atoms. Positional parameters of ligands have been refined with geometric restrictions for bond lengths, bond angles and planarity. The geometry of the final structure was assessed using PROCHECK [35] and WHAT IF [36]. The asymmetric unit of EcDHNA:NP contains residues 1-115 of the DHNA polypeptide chain (122 residues), one NP molecule, and 263 water molecules. The details of structure refinement and the statistics of the final structure are summarized in Table 2.

Atomic coordinates and structure factors have been deposited with the Protein Data Bank under accession codes 2O90.

### Molecular dynamics simulations

The PMEMD module of the Amber molecular dynamics package (version 10) [37] and the Amber ff03 force field [38] were used for the MD simulation of the EcDHNA:DHNP complex. RESP charges for DHNP were derived by using the Antechamber module of Amber 10 with DHNP optimized and its molecular electrostatic potential calculated using the Gaussian program (version 03) [39] at the HF/6-31G* level. The starting coordinate was the crystal structure of EcDHNA:NP with NP replaced by DHNP. All the crystallographic water molecules were removed except the one which is hydrogen bonded to N5 of NP and the amino group of Lys98. The side chain of Lys98 was in the deprotonated form as deemed to be the active form. The protonation and tautomerization states of the histidine residues were assigned taking into account their hydrogen bond interactions in the crystal structure. The default protonation states were used for all of the other amino acids. The system was solvated by a periodic box of TIP3P water molecules that extended at least 12 Å from the protein atoms and neutralized by the addition of Na^+^ ions. The solvent and Na^+^ ions were subjected to energy minimization by 1000 steps steepest descent followed by 1500 steps conjugated gradient minimization while both the protein and DHNP were restrained harmonically with a force constant 50.0 kcal/mol/Å^2^. The MD simulation was run at constant volume for 200 ps to heat up the system from 0 to 300 K while the solutes were restrained in the same way as in the energy minimization, and then at constant temperature and pressure, which were regulated by Langevin dynamics and isotropic position scaling, respectively. The SHAKE algorithm was used to constrain all bond lengths involving hydrogen atoms, permitting a 2-fs time step [40]. The Particle-Mesh-Ewald method was used to evaluate the contribution of long-range electrostatic interactions [41]. A non-bonded pair list cut off 12.0 Å was used, and the list updated every 25 steps. Coordinates were saved every 2 ps. PCA of the MD trajectory was carried out using the program GROMACS 3.2 [42, 43]. The overall translational and rotational motions for all snapshots were removed, and the covariance matrix for all Cα atom fluctuations from their trajectory-averaged values was calculated. The conformational clustering was based on the RMSD for main-chain atoms using the average-linkage algorithm as implemented in the PTRAJ module of Amber 10 as previously described [23].
